# An Eight-Parameter Summary of Clinical Findings Associated with Surgical Intervention in Infants with Necrotizing Enterocolitis Without Radiographic Pneumoperitoneum

**DOI:** 10.3390/children13060776

**Published:** 2026-06-02

**Authors:** Yumeng Liu, Jinpeng Hu, Shuo Zhang, Qingqi Chong, Jinxia Wang, Xiaohui Gong, Zhibao Lv, Qingfeng Sheng

**Affiliations:** 1 Department of General Surgery, Shanghai Children’s Hospital, School of Medicine, Shanghai Jiao Tong University, No. 355, Luding Rd, Shanghai 200062, China; liuyumeng727@163.com (Y.L.); hujinpeng54@163.com (J.H.); sdwczs789@163.com (S.Z.); cqq4411@163.com (Q.C.); zhibao-lv@hotmail.com (Z.L.); 2 Clinical Research Unit, Shanghai Children’s Hospital, School of Medicine, Shanghai Jiao Tong University, No. 355, Luding Rd, Shanghai 200062, China; wangjinxia@shchildren.com.cn; 3 Department of Neonatology, Shanghai Children’s Hospital, School of Medicine, Shanghai Jiao Tong University, No. 355, Luding Rd, Shanghai 200062, China; gongxh@shchildren.com.cn

**Keywords:** necrotizing enterocolitis, surgical intervention, composite indicator

## Abstract

**Highlights:**

**What are the main findings?**

- A higher eight-parameter composite indicator was associated with surgical intervention in infants with NEC without radiographic pneumoperitoneum.
- Peritonitis and abnormal radiographic findings showed the strongest associations with surgical intervention.

**What are the implications of the main findings?**

- These findings describe clinical decision-making patterns and may help inform further research on surgical assessment in NEC without radiographic pneumoperitoneum.

**Abstract:**

**Background**: Determining surgical assessment in infants with necrotizing enterocolitis (NEC) without radiographic pneumoperitoneum remains challenging. This study aimed to describe clinical factors associated with surgical intervention and to retrospectively assess an eight-parameter indicator in this setting. **Methods**: A retrospective study was conducted on infants with Bell stage II–III NEC treated between July 2014 and June 2023. Patients without radiographic pneumoperitoneum were classified into the conservative management group (CON) and surgical intervention group (SUR). Variables considered clinically relevant and suitable for bedside assessment were evaluated for the construction of an exploratory composite indicator. For selected parameters, each item was coded as present or absent, and the total number of positive parameters was calculated for each infant. Clinical characteristics, parameter frequencies, and the distribution of the composite indicator were compared between groups. Receiver operating characteristic (ROC) curve analysis was used descriptively to summarize the apparent discrimination of the composite indicator for observed surgical intervention. **Results**: A total of 115 infants were included; 70 received conservative management and 45 underwent surgical intervention. Eight bedside parameters were selected: endotracheal intubation history, hypotension, peritonitis, radiographic abnormalities, leukopenia, thrombocytopenia, acidosis, and hyponatremia. The SUR group had a higher frequency of several bedside abnormalities and a higher composite indicator score than the CON group. The exploratory composite indicator showed an area under the ROC curve of 0.842 for distinguishing infants who underwent surgical intervention from those managed conservatively. **Conclusions**: Infants with NEC who underwent surgical intervention showed a higher burden of bedside clinical, radiographic, and laboratory abnormalities. This exploratory eight-parameter indicator may provide a practical descriptive summary of disease severity and surgical concern in this challenging clinical setting.

## 1. Introduction

Necrotizing enterocolitis (NEC), a severe intestinal disease, is one of the most common emergencies affecting premature and low birth weight infants. NEC remains a major contributor to neonatal morbidity, affecting nearly 7% of preterm and very low birth weight (VLBW) infants [1,2]. The incidence increases in extremely low birth weight (ELBW) infants, with mortality rates reaching 50.9% [3,4]. NEC predominantly affects preterm and very low birth weight infants, but it can also occur in full-term neonates, often in association with distinct perinatal or underlying clinical conditions [5]. Approximately one-quarter of infants with NEC require surgical intervention [6,7], and timely surgical decision-making represents a significant opportunity to improve patient outcomes [8,9,10]. The presence of pneumoperitoneum is widely recognized as a strong indication for surgical intervention in NEC. However, surgical decision-making is more difficult when radiographic free air is absent. In this setting, infants may still have progressive bowel ischemia, necrosis, or perforation despite the lack of radiographic pneumoperitoneum [11,12,13]. Decisions to escalate from conservative management to surgical intervention, therefore, often depend on a combination of bedside clinical findings, laboratory abnormalities, imaging changes, and the treating team’s judgment. Because these decisions are made in real-world clinical practice rather than according to a single objective marker, the clinical features associated with actual surgical intervention in infants without pneumoperitoneum remain insufficiently defined.

Therefore, this study retrospectively examined infants with Bell stage II–III NEC without radiographic pneumoperitoneum to describe bedside abnormalities associated with observed surgical intervention. We also summarized eight assessable clinical, laboratory, and imaging parameters as an exploratory descriptive composite measure to characterize the overall burden of abnormalities in this population based on our clinical practice.

## 2. Materials and Methods

### 2.1. Patient Enrollment

This study was approved by the Medical Ethics Board of Shanghai Children’s Hospital. We screened infants treated at our institution between July 2014 and June 2023 for necrotizing enterocolitis. Eligible patients met the following inclusion criteria: (1) a diagnosis of Bell stage II or III NEC documented in the medical record; (2) no radiographic evidence of pneumoperitoneum; and (3) availability of clinical, laboratory, and imaging data required for analysis. The diagnosis of NEC was established by the treating clinical team on the basis of systemic manifestations, gastrointestinal signs, laboratory abnormalities, and abdominal radiographic findings consistent with Bell staging.

Exclusion criteria were incomplete medical records, full-term birth, radiographically confirmed pneumoperitoneum before surgery, intestinal stenosis confirmed by surgery or imaging, and withdrawal or refusal of treatment. Patients were then classified according to the definitive treatment received during the index NEC episode as either conservative management (CON) or surgical intervention (SUR). The primary study outcome was surgical intervention during the same NEC episode.

### 2.2. Data Collection

Variables were collected from routinely documented bedside data, previously reported NEC-related factors, and clinical findings considered relevant to surgical assessment in our institution. Demographic information, maternal and perinatal history, pre-NEC exposures, clinical manifestations, radiographic findings, and laboratory results were retrieved from the electronic medical record. First, we collected demographic and clinical data, including gender, gestational age (GA), birth weight (BW), Apgar score, in vitro fertilization (IVF), and congenital heart disease such as patent ductus arteriosus (PDA), patent foramen ovale (PFO), atrial septal defect (ASD), and ventricular septal defect (VSD). We also recorded feeding before the onset of NEC, early postnatal antibiotic use before NEC onset, and supportive care measures, including the administration of curosurf, ibuprofen, corticosteroids, acid-suppressing medications, and umbilical venous catheterization. Additionally, maternal data were collected, encompassing maternal age, singleton or multiple pregnancy, mode of delivery (vaginal or cesarean section), gestational hypertension, preeclampsia, gestational diabetes mellitus, prenatal infections, thyroid disorders, neoplasms, antenatal steroid administration, intrauterine growth retardation (IUGR), placental abnormalities, premature rupture of membranes (PROM), and fetal distress.

Furthermore, clinical information was collected within 96 h after NEC onset in the CON group and from NEC diagnosis until immediately prior to the surgical procedure in the SUR group, capturing the worst results. The data included the following variables: gastric retention, vomiting, abdominal distension, hematochezia, fever, endotracheal intubation, hypotension, tachycardia, bradycardia, increased abdominal circumference, peritonitis, absent bowel sounds, and capillary refill time (CRT) greater than 3 s. Radiographic indicators included pneumoperitoneum, portal venous gas, fixed bowel loop, and severe pneumatosis intestinalis. Laboratory indicators comprised complete blood cell count (CBC), C-reactive protein (CRP), acidosis, biochemical markers, and coagulation profiles. The CBC parameters included white blood cell count (WBC), hemoglobin (HGB), platelet count (PLT), absolute neutrophil count (ANC), absolute lymphocyte count (ALC), absolute monocyte count (AMC), absolute eosinophil count (AEC), and absolute basophil count (ABC). The biochemical markers included alanine aminotransferase (ALT), aspartate aminotransferase (AST), gamma-glutamyl transferase (GGT), albumin, blood urea nitrogen (BUN), creatinine, sodium (Na^+^), potassium (K^+^), and chloride (Cl^−^). The coagulation profiles included prothrombin time (PT), activated partial thromboplastin time (APTT), international normalized ratio (INR), and D-Dimer. Operational definitions were established for selected candidate variables that required explicit clinical criteria, as shown in Table 1.

### 2.3. Statistical Analysis

Statistical analysis was performed using SPSS version 26.0 (IBM SPSS Statistics, Armonk, NY, USA) and graphically presented with GraphPad Prism version 9 (GraphPad Software, San Diego, CA, USA). Continuous variables are presented as medians and interquartile ranges (IQRs). Student’s *t*-test was used for comparisons when continuous variables followed a normal distribution, while the Mann–Whitney U test was applied to compare the non-normally distributed data and the indicator scale between CON and SUR. Categorical information was expressed as counts (percentage), and compared using the Chi-squared test or Fisher’s exact test.

Variables considered clinically relevant and suitable for bedside assessment were further evaluated for the construction of an exploratory composite indicator. Univariable logistic regression was employed for each of the selected parameters. For parameters subsequently included in the composite indicator, each item was coded as 1 when present and 0 when absent, and the total score was calculated as the sum of the selected items. Receiver operating characteristic (ROC) curve analysis was performed to descriptively assess the apparent discrimination of the composite indicator for observed surgical intervention. The area under the curve (AUC) with 95% confidence interval was reported. A *p*-value less than 0.05 was considered statistically significant.

## 3. Results

### 3.1. Cases and Presentation

Between July 2014 and June 2023, a total of 344 infants diagnosed with NEC were treated at Shanghai Children’s Hospital. We excluded cases involving full-term infants (n = 17), Bell Stage I NEC (n = 113), intestinal stenosis confirmed by surgery or imaging (n = 39), infants who declined surgery or treatment (n = 23), incomplete medical records (n = 4), and cases with radiologically confirmed pneumoperitoneum prior to surgery (n = 33). Ultimately, 115 preterm infants were included in the study. Of these, 70 infants received conservative management and 45 underwent surgical intervention. Among the infants who underwent surgical intervention, three received primary peritoneal drainage and the remaining infants underwent laparotomy. Moreover, perforation was identified intraoperatively in 13 cases, and pan-intestinal necrosis was found in 3 cases. We compared baseline characteristics, pre-NEC exposures, clinical manifestations, radiographic findings, and laboratory abnormalities between the conservative management group (CON) and the surgical intervention group (SUR), as detailed below.

### 3.2. Comparison of Baseline Characteristics and Pre-NEC Exposures Between CON and SUR Groups

Initially, we compared baseline characteristics and pre-NEC exposures between the conservative management group (CON) and the surgical intervention group (SUR) in the overall cohort. Infants exposed to maternal infection or PROM showed higher rates of subsequent surgical intervention. These variables were collected as background clinical covariates and were not included in the eight-parameter composite indicator. Due to the limited sample size and the exploratory nature of the analysis, these baseline variables were interpreted cautiously. The rate of confirmed pre-NEC sepsis did not differ significantly between groups. However, when confirmed and suspected sepsis events were considered together, the SUR group showed a higher rate of pre-NEC infectious events than the CON group [29/70 (41.4%) vs. 33/45 (73.3%), *p* < 0.001]. Confirmed sepsis was defined by positive blood culture results, whereas suspected sepsis was defined as empirical antibiotic treatment for at least 72 h based on clinical signs of infection. This discrepancy may be related to the prolonged processing time and limited sensitivity of blood cultures in neonatal practice. Feeding type, achievement of full enteral feeding before NEC onset, and early antibiotic exposure did not show significant differences between groups in this cohort. Overall, baseline characteristics and pre-NEC exposures provided useful clinical context, but they were not sufficient alone to explain the subsequent decision to perform surgical intervention. Detailed demographic, perinatal, and pre-NEC exposure data are shown in Table 2 and Table S1.

### 3.3. Post-NEC Clinical Findings and Surgical Intervention

After NEC onset, clinical manifestations, radiographic findings, and laboratory abnormalities were compared between the conservative management group (CON) and the surgical intervention group (SUR). Compared with the CON group, infants in the SUR group showed higher frequencies of vomiting [8/70 (11.4%) vs. 13/45 (28.9%), *p* = 0.018], abdominal distension [57/70 (81.4%) vs. 45/45 (100.0%), *p* = 0.002], endotracheal intubation [20/70 (28.6%) vs. 28/45 (62.2%), *p* = 0.005], tachycardia [5/70 (7.1%) vs. 20/45 (44.4%), *p* < 0.001], increased abdominal circumference [56/70 (80.0%) vs. 45/45 (100.0%), *p* = 0.001], peritonitis [12/70 (17.1%) vs. 32/45 (71.1%), *p* < 0.001], absent bowel sounds [47/70 (67.1%) vs. 40/45 (88.9%), *p* = 0.008], and prolonged capillary refill time [3/70 (4.3%) vs. 7/45 (15.6%), *p* = 0.047].

Radiographic abnormalities were analyzed as a composite variable, defined as the presence of fixed bowel loops, pneumatosis intestinalis, or portal venous gas. The frequency of abnormal radiographic findings was markedly higher in the SUR group than in the CON group [15/70 (21.4%) vs. 42/45 (93.3%), *p* < 0.001]. Among the laboratory abnormalities, acidosis was more frequent in the SUR group [15/70 (21.4%) vs. 19/45 (42.2%), *p* = 0.017]. Leukopenia, thrombocytopenia, and hyponatremia did not differ significantly between groups. In continuous laboratory variables, the SUR group had lower ALC, ABC, and chloride levels, and higher AST and D-dimer levels. CRP tended to be higher in the SUR group, but the difference did not reach statistical significance. Detailed clinical, radiographic, and laboratory findings are shown in Table 3 and Table S2.

Short-term in-hospital outcomes were also reviewed descriptively to provide clinical context. In the CON group, no infant subsequently converted to surgery during the same NEC episode, and no radiographic perforation was identified during follow-up. Two in-hospital deaths occurred among conservatively managed infants. The in-hospital mortality rate was higher in the SUR group than in the CON group [8/45 (17.8%) vs. 2/70 (2.9%), *p* = 0.013]. Infants who underwent surgery had a greater burden of clinical deterioration and radiographic abnormalities, and the present retrospective study was not designed to determine whether surgical or conservative management resulted in better outcomes. This difference reflects the greater clinical severity of infants who underwent surgical intervention and should not be interpreted as evidence that surgery itself increased mortality. According to the chart review, the two deaths in the CON group were not primarily attributable to NEC progression but were associated with other severe comorbid or systemic conditions. Peritonitis was observed in 12 conservatively managed infants. Among these infants, no in-hospital death, later surgical conversion, or intestinal stenosis was identified. Five of the 12 infants also had abnormal radiographic findings. Despite the presence of both peritonitis and radiographic abnormalities, these infants were managed conservatively without in-hospital death, later surgical conversion, or intestinal stenosis identified during follow-up.

### 3.4. Eight Parameters and Their Association with Surgical Intervention

Candidate variables were compared between the CON and SUR groups. Based on clinical relevance, published evidence, retrospective observations from the present cohort, and multidisciplinary discussion, eight bedside parameters were selected to construct an exploratory composite indicator. These parameters were endotracheal intubation history, hypotension, peritonitis, radiographic abnormalities, leukopenia, thrombocytopenia, acidosis, and hyponatremia.

**Figure 1:**
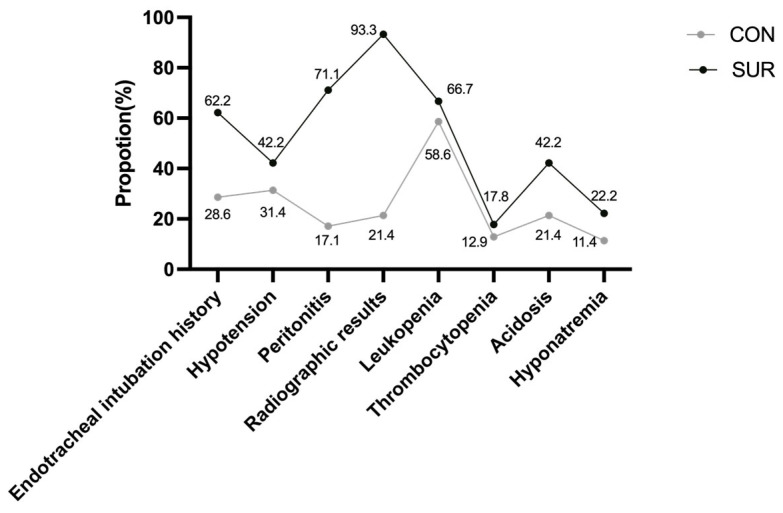
Frequencies of the eight selected parameters in the CON and SUR groups. The proportions of infants with each parameter were compared between groups. All parameters were more frequent in the SUR group.

These eight parameters were selected to summarize different dimensions of clinical deterioration in NEC, including respiratory support, hemodynamic instability, abdominal signs, radiographic abnormalities, hematologic derangement, and metabolic disturbance. The frequencies of the eight selected parameters were compared between the CON and SUR groups. As shown in Figure 1, all eight parameters were more frequently observed in infants who underwent surgical intervention. In particular, endotracheal intubation history, peritonitis, radiographic abnormalities, and acidosis were significantly more common in the SUR group.

Univariable logistic regression was used to describe the association between each parameter and surgical intervention (Table 4). Endotracheal intubation history, peritonitis, radiographic abnormalities, and acidosis were associated with higher odds of surgical intervention. Among these variables, peritonitis and radiographic abnormalities showed the strongest associations.

### 3.5. Exploratory Composite Indicator and ROC Analysis

**Figure 2:**
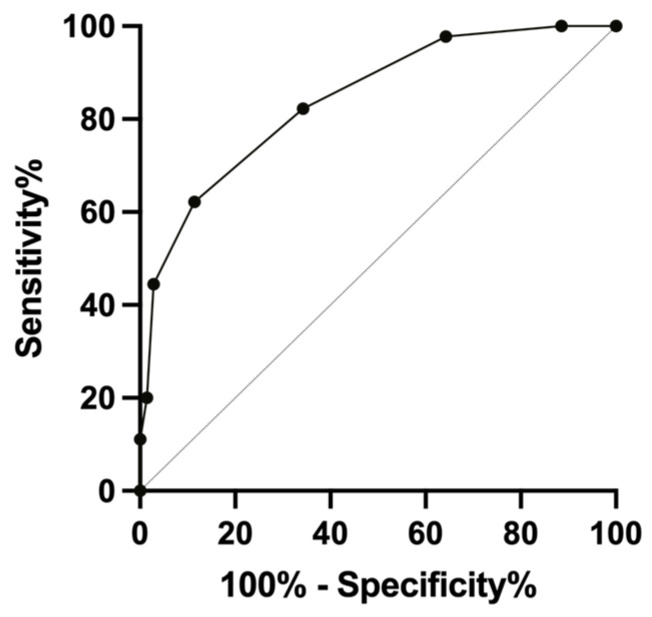
ROC curve of the exploratory eight-parameter composite indicator for observed surgical intervention. The receiver operating characteristic (ROC) curve was used to summarize the apparent discrimination of the exploratory composite indicator for surgical intervention. The y-axis represents sensitivity, and the x-axis represents 1 − specificity. The area under the ROC curve was 0.842 (95% CI, 0.770–0.915). At the exploratory threshold of ≥4 positive parameters, sensitivity, specificity, PPV, and NPV were 62.2%, 88.6%, 77.8%, and 78.5%, respectively.

Each of the eight selected bedside parameters was coded as 1 when present and 0 when absent. The exploratory composite indicator was calculated for each infant by summing these parameters, resulting in a total score ranging from 0 to 8. A higher score indicated a greater burden of clinical deterioration. The CON group had a median score of 2, whereas the SUR group had a median score of 4. A score of 4, corresponding to the median score among infants who underwent surgical intervention, was used as an exploratory reference threshold for descriptive assessment. ROC curve analysis was performed to summarize the discriminative performance of the composite indicator for observed surgical intervention. In the descriptive ROC analysis, the exploratory composite indicator showed an apparent AUC of 0.842 (95% CI, 0.770–0.915) for observed surgical intervention (Figure 2). At the exploratory reference threshold of ≥4 positive parameters, the sensitivity was 62.2%, the specificity was 88.6%, the positive predictive value was 77.8%, and the negative predictive value was 78.5%.

## 4. Discussion

In this retrospective study, we analyzed infants with NEC without radiographic pneumoperitoneum and summarized an exploratory eight-parameter bedside composite indicator, including history of endotracheal intubation, hypotension, peritonitis, abnormal radiographic findings, leukopenia, thrombocytopenia, acidosis, and hyponatremia.

Among the eight components, peritonitis and abnormal radiographic findings showed the strongest associations with surgical intervention. These findings are clinically plausible and consistent with current surgical practice, because both abdominal signs and worsening radiographic abnormalities often reflect advanced bowel injury [14,15]. Although no in-hospital deaths, later conversion to surgery, or perforation were observed among conservatively managed infants with peritonitis in our cohort, this finding should not be interpreted as proof that the non-operative decision was necessarily optimal in every case. Potential incorporation bias may exist because peritonitis and radiographic abnormalities likely contributed to the decision to operate. Thus, these associations may reflect clinical decision-making rather than independent prognostic value. The remaining six variables were retained because they capture important aspects of disease progression that are readily assessable in routine care. Endotracheal intubation history and hypotension reflect systemic deterioration and hemodynamic instability, whereas leukopenia and thrombocytopenia indicate hematologic and inflammatory compromise [16]. Acidosis and hyponatremia were included as markers of metabolic disturbance [17,18]. Although not all parameters were statistically significant, they were included to summarize different dimensions of clinical deterioration.

The exploratory eight-parameter composite indicator was used to summarize the number of concerning findings present in each infant. One clinical value of this analysis lies in the setting where radiographic pneumoperitoneum is absent but disease progression remains concerning. In such cases, the decision to escalate is often based on the combined interpretation of clinical signs, laboratory abnormalities, and imaging findings rather than a single indicator. Our analysis may help summarize these routinely available findings and support bedside assessment of overall clinical severity. Therefore, it should not be used independently to guide surgical intervention or to replace individualized clinical judgment. In particular, a higher number of positive parameters may indicate greater clinical severity, but it does not by itself establish that surgery is definitely required. Surgical decision-making in NEC remains complex and depends on the dynamic clinical course, the infant’s overall condition, institutional practice, and the judgment of the treating team.

Previous studies have also explored predictors of severe NEC or the need for surgery, either by identifying individual associated factors or by developing composite scoring systems [14,19,20]. Tepas et al. emphasized metabolic derangement trajectories [21], whereas Munaco et al. integrated multiple clinical and radiographic variables into a broader predictive framework [22,23]. Our study adds a single-center experience, focusing on routinely available bedside parameters associated with actual surgical intervention in infants without radiographic pneumoperitoneum. However, comparisons across studies should be interpreted cautiously because of differences in patient selection, outcome definitions, and local treatment strategies.

Severe NEC, especially when surgery is ultimately required, has been associated with worse neurodevelopmental outcomes [24]. However, in clinical practice, the timing of surgical intervention is usually determined as the best available choice based on the infant’s current short-term clinical presentation. Whether earlier intervention improves long-term neurodevelopmental outcomes remains uncertain. Future prospective studies should therefore evaluate not only short-term decision-making and survival, but also later developmental outcomes.

This study has several limitations. First, it was a retrospective single-center study with a relatively small sample size. Second, the primary outcome reflects real-world clinical decision-making rather than a definitive gold standard for surgical necessity and may limit generalizability. More research is needed to establish a standardized system for infants with NEC without radiographic pneumoperitoneum.

## 5. Conclusions

In this single-center retrospective cohort, surgical intervention in infants with NEC without radiographic pneumoperitoneum was associated with a higher burden of clinical, radiographic, and laboratory abnormalities. The exploratory eight-parameter composite indicator may provide a descriptive summary of clinical decision-making. Further multicenter studies are needed to refine the assessment framework for this patient population.

## Figures and Tables

**Table 1:** Operational definitions of candidate variables.

Definitions	Criteria
Early postnatal antibiotic exposure	Systemic antibiotic use within 72 h after birth and before NEC onset
Endotracheal intubation history	Invasive mechanical ventilation history
Hypotension	MAP < gestational age or on any pressor
Peritonitis	Any sign of abdominal erythema and swelling, abdominal tenderness, or abdominal rigidity
Increased abdominal circumference	Increase in abdominal circumference by ≥2 cm during the assessment window
Radiographic abnormalities	Fixed bowel loops, pneumatosis, or portal venous gas
Leukopenia	Absolute leukocyte count ≤ 8 × 10^9^/L
Thrombocytopenia	Platelet count < 50,000/mm^3^
Acidosis	pH < 7.25 or receiving bicarbonate/THAM
Hyponatremia	Sodium < 130 mEq/L

NEC: necrotizing enterocolitis, MAP: mean arterial pressure, THAM: Tris(hydroxymethyl)aminomethane.

**Table 2:** Baseline characteristics and pre-NEC exposures.

Findings	CON (n = 70)	SUR (n = 45)	*p* Value
Male, n (%)	44 (62.9%)	28 (62.2%)	0.945
GA, w, mean ± SD	31.3 ± 2.7	31.1 ± 3.4	0.672
BW, g, median (IQR)	1505 (1272–1910)	1450 (1243–1985)	0.998
1 min Apgar Score,	8 (7–9)	8 (8–9)	0.463
median (IQR)			
PDA, n (%)	39 (55.7%)	20/44 (45.5%)	0.286
PFO, n (%)	42 (60%)	24/44 (54.4%)	0.566
ASD, n (%)	46 (65.7%)	34/44 (77.3%)	0.189
VSD, n (%)	4 (5.7%)	2/44 (4.5%)	1
Sepsis before NEC, n (%)	8 (11.4%)	4 (8.9%)	0.762
MOM or DHM only, n (%)	12/68 (17.6%)	4 (8.9%)	0.191
Mixed MOM DHM and formula, n (%)	23/68 (33.8%)	15 (33.3%)	0.957
Formula only, n (%)	31/68 (45.6%)	25 (55.6%)	0.3
Not fed, n (%)	1/68 (1.5%)	1 (2.2%)	1
Full enteral feeding before NEC onset, n (%)	34/65 (52.3%)	23 (51.1%)	0.908
Antibiotics, 1–3 d, n (%)	5/65 (7.7%)	2/40 (5%)	0.706
Antibiotics, Beyond 3 d, n (%)	47/65 (72.3%)	30/40 (75%)	0.762
No use of antibiotics, n (%)	13/65 (20%)	6/40 (15%)	0.518
Advanced antibiotics, n (%)	11/65 (16.9%)	5/40 (12.5%)	0.54
Curosurf, n (%)	22 (33.8%)	18 (45%)	0.253
Ibuprofen, n (%)	7 (10.8%)	1 (2.5%)	0.151
Corticosteroids, n (%)	4 (6.2%)	6 (15%)	0.175
Acid-suppressing medications, n (%)	1 (1.5%)	0	1
Umbilical venous catheterization, n (%)	15 (23.1%)	10 (25%)	0.822
Age of NEC onset, d	14 (9–25)	15 (7–30)	0.866
median (IQR)			
Maternal age, y, mean ± SD	31.4 ± 5.1	30.4 ± 4.0	0.236
Prenatal infection, n (%)	10 (14.3%)	15 (33.3%)	0.016
PROM, n (%)	17 (24.3%)	23 (51.1%)	0.003
Fetal Distress, n (%)	9 (12.9%)	8 (17.8%)	0.468

GA: gestational age, BW: birth weight, PDA: patent ductus arteriosus, PFO: patent foramen ovale, ASD: atrial septal defect, VSD: ventricular septal defect, MOM: mother’s own milk, DHM: donor human milk, PROM: premature rupture of membranes. Data are presented as n (%) or n/N (%) where appropriate. For variables with missing data, N indicates the number of evaluable cases.

**Table 3:** Comparison between the CON group and the SUR group of clinical data.

Findings	CON (n = 70)	SUR (n = 45)	*p* Value
Gastric retention, n (%)	45 (64.3%)	36 (80%)	0.072
Vomiting, n (%)	8 (11.4%)	13 (28.9%)	0.018
Abdominal distension, n (%)	57 (81.4%)	45 (100%)	0.002
Hematochezia, n (%)	34 (48.6%)	27 (60%)	0.231
Fever, n (%)	18 (25.7%)	12 (26.7%)	0.910
Endotracheal intubation, n (%)	20 (28.6%)	28 (62.2%)	0.005
Hypotension, n (%)	22 (31.4%)	19 (42.2%)	0.238
Tachycardia, n (%)	5 (7.1%)	20 (44.4%)	<0.001
Increased abdominal circumference, n (%)	56 (80%)	45 (100%)	0.001
Peritonitis, n (%)	12 (17.1%)	32 (71.1%)	<0.001
Absent bowel sounds, n (%)	47 (67.1%)	40 (88.9%)	0.008
CRT > 3 s, n (%)	3 (4.3%)	7 (15.6%)	0.047
Radiologic abnormalities, n (%)	15 (21.4%)	42 (93.3%)	<0.001
WBC (×10^9^/L), median (IQR)	7.40 (5.02–11.8)	6.52 (3.63–10.3)	0.612
HGB (g/L), median (IQR)	120 (96.5–154)	111 (89.0–146)	0.465
PLT (×10^9^/L), median (IQR)	221 (108–334)	221 (134–326)	0.873
ANC (×10^9^/L), median (IQR)	2.78 (1.55–5.75)	3.47 (1.62–7.37)	0.856
ALC (×10^9^/L), median (IQR)	3.45 (1.99–4.60)	1.95 (0.99–2.81)	<0.001
AMC (×10^9^/L), median (IQR)	1.06 (0.57–1.65)	0.78 (0.41–1.57)	0.339
AEC (×10^9^/L), median (IQR)	0.31 (0.11–0.64)	0.06 (0.02–0.15)	0.050
ABC (×10^9^/L), median (IQR)	0.04 (0.02–0.08)	0.02 (0.00–0.05)	0.016
CRP (mg/L), median (IQR)	18.0 (5.00–47.5)	32.0 (10.0–65.5)	0.052
Lactic acid (mmol/L), median (IQR)	2.00 (0.90–3.30)	2.10 (1.50–2.83)	0.833
ALT (U/L), median (IQR)	9.50 (6.00–29.5)	10.0 (7.00–16.3)	0.743
AST (U/L), median (IQR)	32.0 (22.0–48.0)	51.0 (28.8–69.8)	0.023
GGT (U/L), median (IQR)	92.0 (69.0–126)	71.5 (37.5–114)	0.071
Cl^−^ (mmol/L), median (IQR)	104 (101–106)	101 (96.3–105)	0.018
D-Dimer (mg/L, FEU), median (IQR)	2.27 (1.28–4.22)	4.15 (2.14–8.47)	0.003
Acidosis, n (%)	15 (21.4%)	19 (42.2%)	0.017
Hyponatremia, n (%)	8 (11.4%)	10 (22.2%)	0.12

CRT: capillary refill time. WBC: white blood cell, HGB: hemoglobin, PLT: platelet, ANC: absolute neutrophil count, ALC: absolute lymphocyte count, AMC: absolute monocyte count, AEC: absolute eosinophil count, ABC: absolute basophil count, CRP: C-reactive protein, ALT: ala-nine aminotransferase, AST: aspartate aminotransferase, GGT: gamma-glutamyl transferase, Leukopenia was defined as a white blood cell count ≤ 8 × 10^9^/L. Thrombocytopenia was defined as a platelet count < 50,000/mm^3^. Acidosis was defined as pH < 7.25 or treatment with bicarbonate or THAM. Hyponatremia was defined as serum sodium < 130 mEq/L.

**Table 4:** Univariable associations between the eight parameters and surgical intervention.

Characteristics	Univariable OR	95% CI	*p* Value
Endotracheal intubation history	4.12	1.86–9.12	*
Hypotension	1.60	0.73–3.47	0.240
Peritonitis	11.9	4.86–29.1	*
Radiographic abnormalities	51.3	13.9–189	*
Leukopenia	1.42	0.65–3.09	0.384
Thrombocytopenia	1.47	0.52–4.13	0.470
Acidosis	2.68	1.18–6.10	0.019
Hyponatremia	2.21	0.80–6.13	0.126

OR: odds ratio, CI: confidence interval. * *p* < 0.001.

## Data Availability

The data that support the findings of this study are available from the corresponding author. The data are not publicly available due to the policy of Shanghai Children’s Hospital.

## Supplementary Materials

The following supporting information can be downloaded at: https://www.mdpi.com/article/10.3390/children13060776/s1. Table S1. Supplemental baseline characteristics and pre-NEC exposures. Table S2. Supplemental comparison of clinical data.

## Abbreviations

The following abbreviations are used in this manuscript:

NEC	Necrotizing enterocolitis
CON	Conservative management group
SUR	Surgical intervention group
ROC	Receiver operating characteristic
AUC	Area under the receiver operating characteristic curve
PPV	Positive predictive value
NPV	Negative predictive value
