# Supplementary material for: An Eight-Parameter Summary of Clinical Findings Associated with Surgical Intervention in Infants with Necrotizing Enterocolitis Without Radiographic Pneumoperitoneum
Source: Children (Basel). 2026 Jun 2;13(6):776. doi: 10.3390/children13060776 (PMC13297530; doi:10.3390/children13060776)
Supplement: Supplementary file 1 [file children-13-00776-s001.zip › children-4326197-supplementary.pdf]

Supplemental table S1. Supplemental baseline characteristics and pre-NEC exposures.

**Findings**	**CON (n=70)**	**SUR (n=45)**	***P* value**
5 min Apgar Score, median (IQR)	9(8–9)	9(8–10)	0.452
Complex CHD, n (%)	3(4.3%)	0	0.283
Digestive system malformations, n (%)	2(2.9%)	0	0.522
Urinary or nervous system malformations, n (%)	2(2.9%)	0	0.522
Bronchopulmonary dysplasia, n (%)	17(24.3%)	5/44(11.4%)	0.089
Gestational hypertension, n (%)	9(12.9%)	8(17.8%)	0.468
Preeclampsia, n (%)	10(14.3%)	8(17.8%)	0.615
Antenatal steroids, n (%)	16(22.9%)	11(24.4%)	0.845
IUGR, n (%)	4(5.7%)	2(4.4%)	1
Placental abnormalities, n (%)	6(8.6%)	0	0.08

IUGR: intrauterine growth retardation.

Data are presented as n (%) or n/N (%) where appropriate. For variables with missing data, N indicates the number of evaluable cases.

Supplemental table S2. Supplemental comparison of clinical data.

Findings	CON (n=70)	SUR (n=45)	*P* value
Bradycardia, n (%)	4(5.7%)	3(6.7%)	1
pH, median (IQR)	7.32(7.25–7.39)	7.29(7.20–7.37)	0.156
Albumin (g/L), median (IQR)	31.8(27.7–34.5)	30.2(26.1–32.5)	0.071
BUN (mmol/L), median (IQR)	4.40(2.80–6.10)	5.35(3.40–7.40)	0.446
Creatinine(μmol/L), median (IQR)	31.0(25.3–42.5)	34.0(28.5–42.5)	0.134
Na^+ (mmol/L), median (IQR)	136(134–138)	134(129–137)	0.115
K^+ (mmol/L), median (IQR)	4.50(3.90–5.05)	4.70(4.20–4.95)	0.495
Cl^- (mmol/L), median (IQR)	104(101–106)	101(96.3–105)	0.018
PT (s), median (IQR)	14.5(12.4–16.3)	14.9(13.6–17.5)	0.334
APTT (s), median (IQR)	57.5(46.2–66.5)	52.4(44.0–67.7)	0.953
INR, median (IQR)	1.25(1.10–1.44)	1.36(1.18–1.57)	0.377
D-Dimer (mg/L, FEU), median (IQR)	2.27(1.28–4.22)	4.15(2.14–8.47)	0.003

BUN: blood urea nitrogen, PT: prothrombin time, APTT: activated partial thromboplastin time, INR: interna-tional normalized ratio.
